# Feasibility of smartphone colorimetry of the face as an anaemia screening tool for infants and young children in Ghana

**DOI:** 10.1371/journal.pone.0281736

**Published:** 2023-03-03

**Authors:** Thomas Alan Wemyss, Miranda Nixon-Hill, Felix Outlaw, Anita Karsa, Judith Meek, Christabel Enweronu-Laryea, Terence S. Leung

**Affiliations:** 1 Department of Medical Physics and Biomedical Engineering, Malet Place Engineering Building, University College London, London, United Kingdom; 2 Neonatal Care Unit, EGA Wing, University College London Hospitals, London, United Kingdom; 3 Department of Child Health, University of Ghana Medical School, College of Health Sciences, University of Ghana, Accra, Ghana; Wachemo University, INDIA

## Abstract

**Background:**

Anaemia affects approximately a quarter of the global population. When anaemia occurs during childhood, it can increase susceptibility to infectious diseases and impair cognitive development. This research uses smartphone-based colorimetry to develop a non-invasive technique for screening for anaemia in a previously understudied population of infants and young children in Ghana.

**Methods:**

We propose a colorimetric algorithm for screening for anaemia which uses a novel combination of three regions of interest: the lower eyelid (palpebral conjunctiva), the sclera, and the mucosal membrane adjacent to the lower lip. These regions are chosen to have minimal skin pigmentation occluding the blood chromaticity. As part of the algorithm development, different methods were compared for (1) accounting for varying ambient lighting, and (2) choosing a chromaticity metric for each region of interest. In comparison to some prior work, no specialist hardware (such as a colour reference card) is required for image acquisition.

**Results:**

Sixty-two patients under 4 years of age were recruited as a convenience clinical sample in Korle Bu Teaching Hospital, Ghana. Forty-three of these had quality images for all regions of interest. Using a naïve Bayes classifier, this method was capable of screening for anaemia (<11.0g/dL haemoglobin concentration) vs healthy blood haemoglobin concentration (≥11.0g/dL) with a sensitivity of 92.9% (95% CI 66.1% to 99.8%), a specificity of 89.7% (72.7% to 97.8%) when acting on unseen data, using only an affordable smartphone and no additional hardware.

**Conclusion:**

These results add to the body of evidence suggesting that smartphone colorimetry is likely to be a useful tool for making anaemia screening more widely available. However, there remains no consensus on the optimal method for image preprocessing or feature extraction, especially across diverse patient populations.

## Introduction

Anaemia is estimated to be the 14th leading cause of disease in the world by disability-adjusted life years [1]. The condition is characterized by a reduced concentration of functional haemoglobin in the blood, which results in a lessened ability to transport oxygen around the body. The most common cause of anaemia globally is iron-deficiency [2]; but other conditions, such as blood loss, malaria (via parasite induced haemolysis [3]), and sickle-cell disease also contribute. Even in regions in which malaria is a significant contributor to anaemia development, iron-deficiency is still present in around 40% of cases of paediatric anaemia [4]. Uncertainties about the exact diagnostic criteria to use for iron-deficiency anaemia may delay diagnosis and treatment [5], and such a delay in diagnosis may occur regardless of the exact type of anaemia; in the case of sickle cell anaemia, patients may only present to clinic when in acute pain due to vessel occlusion by sickled cells [6]. Late diagnosis may worsen patient outcomes through delaying treatment.

Developmental outcomes can be adversely affected by paediatric anaemia; iron deficiency anaemia is associated with a reduced cognitive development index, which generally improves significantly with treatment [7]. However, it has been suggested that early treatment is necessary if good long-term outcomes are to be achieved, as iron deficiency may otherwise lead to lasting changes in neuronal physiology and biochemistry [8]. Early treatment is also likely to be useful to avoid more severe, rarer, effects. For example, untreated anaemia may also result in central sinovenous thrombosis [9] or ischaemic stroke [10], especially in children between 6 and 18 months of age. In the local context of Ghana, where this data was collected, sickle cell anaemia has a greater prevalence than in cooler climates. Sickle cell anaemia can also be associated with renal dysfunction [11, 12] or even ocular manifestations of disease [13]. Given the clear benefit of early treatment and diagnosis, it is important to understand local and global factors which contribute to it not taking place.

### Current techniques for anaemia screening

A range of factors, such as education, diet, and access to healthcare make untreated iron-deficiency more common in certain regions [14, 15]. Local factors (such as geographical scale) may make traditional tests for anaemia harder to access in certain regions. Laboratory based tests, which tend to be the cheapest per-test at scale, have a requirement for the sample to be transported between the clinic and the laboratory. This introduces a latency between test sample collection, and the time that the results are available to inform clinical care. Such a delay might mean that a patient needs to make two visits to hospital, one for a blood sample and another to receive their results, or may even mean that a seriously ill patient occupies a hospital bed awaiting diagnosis and treatment. The clinical desire for prompt results to overcome this helped a portable device, the HemoCue, gain clinical acceptance during the 1980s [16]; the HemoCue can analyse a finger-prick blood sample to provide results in the healthcare setting within one minute. However, the HemoCue has a significant upfront cost, as well as relatively high ongoing costs, and consumables with relatively short shelf lives of approximately 2 years. When compounded with the fact that the HemoCue still requires a blood sample (albeit a simpler finger-prick blood sample), it remains constrained to healthcare environments.

Studies of hospital access in Ghana have shown that distance from the healthcare clinic is the largest factor that impacts whether patients access healthcare [17], and that access to hospitals outside urban areas is often challenging [18]. At-home screening for disease is likely to be beneficial in overcoming this. It has been suggested that haemoglobin colour cards to aid visual judgement of anaemia might be used for this purpose, but these can suffer from low specificity (below 50% in some studies, even when judged by experienced observers [19]). Consequently, there has been a drive towards developing an objective, automated, at-home screening system.

### Smartphone colorimetry for anaemia screening

A natural technology for at home screening for a compound such as haemoglobin with a characteristic absorption pattern within the visible wavelengths is the smartphone camera. Smartphone imaging is relatively low risk (no ionizing radiation, no incision or laceration needed), and smartphones are increasingly popular globally. In the case of jaundice, where the raised blood concentration of bilirubin leads to visible yellowing of the skin, smartphone colorimetry has been carried out successfully without the need to take any blood sample [20–24]. It is therefore unsurprising that there has been extensive prior research into using smartphones as a tool with which to screen for anaemia (Table 1).

**Table 1:** Quantitative comparison of a selection of imaging methods for screening for anaemia.

Study	Main Ethnicity, Age (N)	Preprocessing	Feature	Region	Sens./Spec.
Kent et al. 2000 [25]	Mixed Adults (29)	Controlled lighting	Human vision	Lower eyelid	31% / 89%
Suner et al. 2007 [26]	Adult Mixed (63)	White reference	All pixel values	Lower eyelid	69% / 72%
Collings et al. 2016 [27]	Mostly European Adults (47)	Camera whitebal.	Erythema index	Lower eyelid	74% / 71%
Chen et al. 2016 [28]	Chinese Adults (100)	Camera whitebal.	Multiple	Lower eyelid	78% / 83%
Bevilacqua et al. 2016 [29]	Mostly European Adults (77)	Controlled lighting	a* (L*a*b*)	Lower eyelid	100% / 82%
Irum et al. 2016 [30]	Pakistani Adults (77)	Histogram eq.	Multiple	Lower eyelid	88% / 81%
Hasan et al. 2017 [31]	North American Adults (30)	Camera whitebal.	Pixel histogram	Fingertip	100%^⊤^ / 100%^⊤^
Chen and Miaou 2017 [32]	Chinese Adults (100)	Camera whitebal.	Red pixel value	Lower eyelid	76% / 81%
Mannino et al. 2018 [33]	Mixed Adults (100)	Camera whitebal.	All pixel values	Fingernail	92% / 76%
Dimauro et al. 2018 [34]	Mostly European Adults (113)	Controlled lighting	a*, b*, G	Lower eyelid	
Lobbes et al. 2019 [35]	Mostly White Adults (67)	White-reference	Blue colour	Sclera	78% / 50%
Kasiviswanathan et al. 2020 [36]	Indian Adults (135)	None	Multiple	Lower eyelid	
Jain et al. 2020 [37]	Mixed Adults (99)	Controlled lighting	Neural network	Lower eyelid	99%* / 95%*
Narayan et al. 2020 [38]	N.A.	Histogram eq.	Multiple	Conj, nail, tongue	
Suner et al. 2021 [39]	Mixed Adults (344)	Camera whitebal.	Multiple	Lower eyelid	73% / 73%
*Wemyss et al. 2022*	*Ghanaian Children (43)*	*Multiple*	*Multiple*	*Conj, lip, sclera*	*93% / 90%*
Mean (excluding this)					78% / 82%

Sensitivity (“Sens.”) and specificity (“Spec.”) for several papers investigating similar methodology. Unless otherwise marked, values reported are for a 11.0 g/dL cutoff to define anaemia.

^⊤^ indicates the cutoff was 10.0 g/dL.

A blank value indicates either that no comparable results were disclosed, or that the paper was methodological only, and hence results were not present.

* indicates that it is not clear whether there was data leakage from the training set to the testing set due to the augmentation strategy used by the authors.

Conj stands for lower eyelid conjunctiva

whitebal. stands for for white balancing

There remain several unknown parameters. A key one is which area of the body to image—the areas which can be used are limited by the presence of skin pigmentation within the melanocytes [40]. This pigmentation occludes the blood chromaticity, which could introduce a prediction error that depends on skin pigmentation. The potential of error due to skin pigmentation can be avoided by analysing areas of the body which have reduced numbers of melanocytes: the bed beneath the nails, the sclera (white of the eye), the lower palpebral conjunctiva (the membrane beneath the lower eyelid) and the mucosal membrane of the mouth adjacent to the lower lip. Several of these regions have been used previously with promising results in specific populations, and are summarized in Table 1. However, at the time of writing, none of these methods have approval from the United States of America Food and Drug Administration or medical device approval for other markets, and there remains some uncertainty about the optimum algorithm to use, especially in populations with greater pigmentation.

In fact, Table 1 demonstrates there is no clear consensus on how to overcome several key challenges. The first challenge is the fact that different environments have different lighting. This means that the chromaticities of the captured image would vary depending on where the image is taken. There are two possible solutions: (1) to account for varying ambient lighting, or (2) to control the ambient lighting. In order to account for varying lighting, some studies use the automatic white-balancing features within the camera/phone [27, 28], but it remains uncertain how methods which rely on manufacturer-specific inbuilt camera white balancing might perform when deployed on a variety of different phone models. In screening for other conditions, some success has been obtained by using “known” white-balancing algorithms, such as sclera white balancing [41]. Such a technique allows for the same white balancing to be performed regardless of the camera/phone software. However, white balancing will be naturally limited by the existence of metamerism and low colour-rendering-index lighting. To overcome this, other work attempts to control the ambient lighting itself, for example by blocking it out physically using specially manufactured shields [29]). However, this may increase the cost and difficulty of distributing a system widely. To overcome this, ambient subtraction (as introduced by [20, 42]) is an appealing technique—it allows estimation of the image chromaticities under a fixed illuminant (the camera flash alone), but without the need for hardware to physically block the light. However, ambient subtraction has not yet been used in anaemia screening. The present study will therefore compare the performance of white balancing against the performance of ambient subtraction.

Once the image has been preprocessed, the second challenge is to determine which feature to extract and analyse, and where on the image from which to extract it. The fingernails are a promising region of interest but may lead to excluding a large number of patients from analysis. For example [33], stated that patients were excluded “if their images showed fingernail beds that were obscured or discoloured due to leukonychia, nailbed injury, nail polish, [or] darkening due to medication”. Even healthy female children in India were found to have around a 42% prevalence of nail opacities [43] (although it should be noted that only a single clear nail is required for the analysis). In cultures in which Henna (*Lawsonia inermis*) or nail varnish are used for colouring of fingernails, this may lead to exclusions which are imbalanced towards excluding particularly vulnerable groups, such as pregnant women. This effect is compounded by the fact that—in rare cases in G6PD enzyme deficient individuals—Henna application can actually be the cause of haemolytic anaemia [44]. Consequently, there remains some utility in attempting colorimetry other regions: the palpebral conjunctiva of the lower eyelid has been widely investigated in adult populations (details in Table 1). Images of the eyelid often include the sclera due to their physical proximity, and the sclera itself is a potential target for analysis, because it has a network of conjunctival vasculature providing the opportunity to measure blood chromaticity with little filtering from superficial tissue. If collecting additional images is possible, the mucosal membrane of the mouth adjacent to the lower lip provides a further potential target for colorimetric analysis, with dense vasculature. This lower lip region has not previously been widely studied for the purposes of anaemia screening.

In order to use any region for colorimetric screening, a metric to represent the blood chromaticity (and by proxy, the blood haemoglobin concentration) is extracted. Intuitively, given the red colour of (oxy)-haemoglobin, it seems likely that *r*-chromaticity (the ratio of the red channel of a pixel to the total signal at that pixel) is likely to prove a useful metric. Several other papers use alternative chromaticity spaces (such as CIE 1976 L*a*b* in [29]), or ratios of different colour channels within the image (such as erythema index, [45]). All of these techniques have the benefit of being relatively explainable—it is intuitive that “a greater haemoglobin concentration” would relate to “more redness”. This is preferred because reviews suggest that explainable artificial intelligence tools are more likely to be accepted [46]. Other techniques, such as neural networks, use all (or a random sample) of the pixel values within the region of interest, in conjunction with neural networks or high numbers of parameters in linear models. Although these have given very promising results, for the present research, only explainable redness metrics will be examined.

### Aims

The present study aims to investigate and compare methods for ambient lighting correction (ambient subtraction; sclera white balancing) and image redness measures (erythema index, *r* chromaticity, g-chromaticity, CIE 1976 a*b* chromaticities). This research is carried out in an understudied population of infants and young children in Ghana. In contrast to previous works, which have primarily focussed on a single region of interest, this paper further aims to investigate whether screening performance might be further increased through linear combinations of colorimetric metrics from additional regions of interest: the lower eyelid and the lower lip.

## Materials and methods

**Fig 1:**
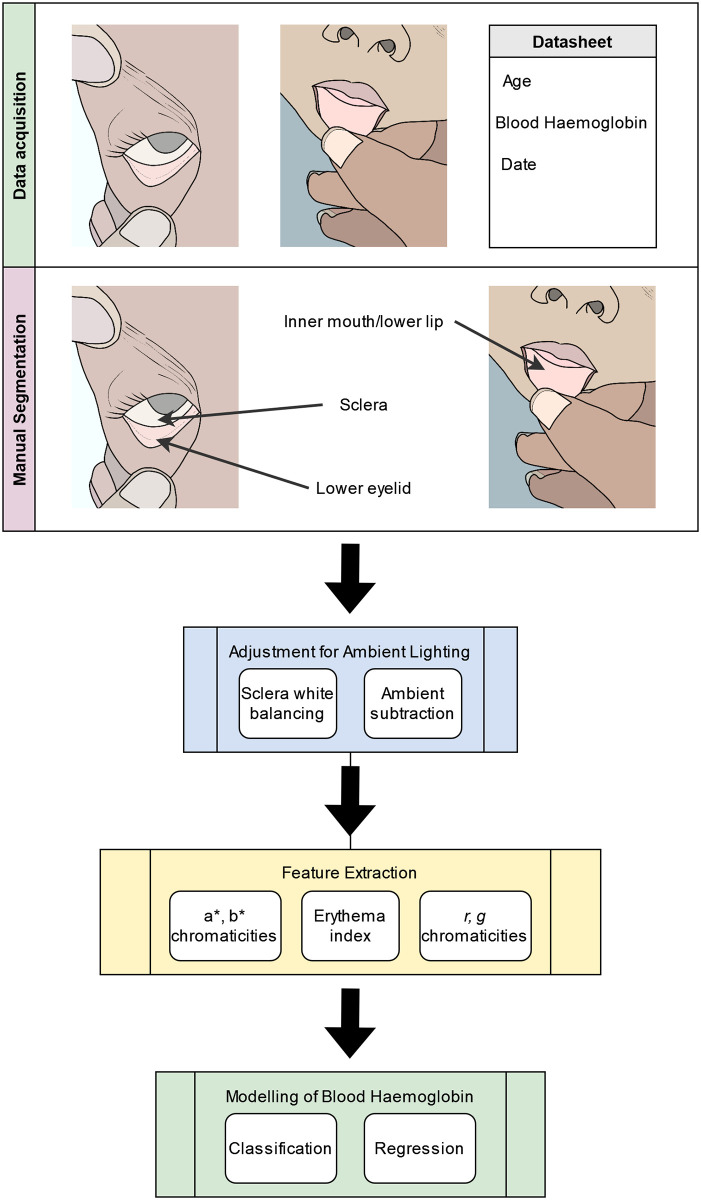
Summary of methods. A flow chart of the overall analysis pipeline used for the present research.

This section discusses data collection, image pre-processing, feature extraction, and subsequent analysis and validation. This process is summarized in Fig 1.

### Data collection

Between February and April 2018, a convenience sample of 62 children aged between birth and 59 months of age was recruited in Korle Bu Teaching Hospital, Ghana. Recruitment was carried out in accordance with University College London Ethics application 4050/003, and proposals submitted to the Ethical and Protocol Review Committee of the College of Health Sciences, University of Ghana as CHS-Et/M.5-P1.11/2017–2018. Written consent was given by the parents of the participants. Additional information regarding the ethical, cultural, and scientific considerations specific to inclusivity in global research is included in the (S1 Checklist).

Participants were only recruited when they were clinically stable, where a blood test was requested by their attending doctor as part of their standard clinical care, and when urgent care would not be delayed by the acquisition of the images for this study. Participants were excluded if they were receiving oxygen, had received a blood transfusion in the last 24 hours, or were otherwise deemed to be very ill or unstable. Participants were excluded when conditions such as uveitis or gingivitis that affected the colouration of the imaged mucosa were recorded. Before participation, the research nurse/doctor explained the purpose of the study, and answered any questions which the parent or child raised. After all questions were answered, parents were provided with written consent forms prior to image acquisition.

For each participant, blood haemoglobin concentration was measured with a HemoCue Hb 301 point-of-care anaemia screening device (HemoCue AB, Ängelholm, Sweden) in accordance with the manufacturer’s guidelines, after which they were immediately imaged by a trained clinician. The imaging was carried out using the back-facing camera on a Samsung Galaxy S8 smartphone with a custom mobile application which captured and stored raw, lossless (Adobe Digital Negative) camera images. Using raw images means that there was minimal post-sensor processing. There were 3 pairs of images taken: (1) an image of the sclera and surrounding skin, (2) an image of the folded-over lower eyelid, and (3) an image of the lower lip. Each pair of images consisted of an image taken with the smartphone camera flash turned on, and another taken automatically afterwards with the smartphone camera flash turned off. The flash was diffused using a polymer diffuser in order to ensure the flash brightness fell within safe limits.

### Image analysis

Images were analysed using MATLAB R2022b (The MathWorks, Inc., USA). For each participant, three regions of interest (ROIs) were manually segmented: the sclera, the lower palpebral conjunctiva, and the lower lip. These regions are shown in Fig 1. Segmentation was carried out by a single observer who was not aware of the age, blood haemoglobin, or other demographic details of the patient at the time of segmentation. The images were then pre-processed in order to account for varying ambient lighting conditions, after which candidate measures to correlate against blood haemoglobin concentration were extracted.

#### Image pre-processing

After Bayer demosaicing the RAW image to convert it into camera native RGB, any pixels where any R, G, or B component was nearly saturated (>99%) were removed. A device specific calibration was used to convert images from the camera native RGB colourspace into the CIE XYZ colourspace [42]. This was then converted into the linearized Reference Output Medium Metric (ROMM) RGB colourspace prior to adjustment for varying ambient lighting. The ROMM colourspace was chosen as it is a wide gamut colourspace which represents most colours in the natural world. Any pixels which lay outside the ROMM colourspace were discarded automatically. Two approaches were investigated in order to obtain consistent image measures in varying ambient lighting.

**(1) Ambient subtraction** Ambient subtraction was carried out as per [20]. The red (R), green (G), and blue (B) values for the region of interest were calculated using the formula in Eq 1 for each image channel (*I*), where *I*_*flash*+*ambient*_ was the selected pixels within the region of interest of the image taken with the camera flash and ambient lighting, and *I*_*ambient*_ was the selected pixels within the same region of interest of the image taken under ambient lighting alone.

$$\begin{array}{c} I_{flash}=\text{median}\left(I_{flash+ambient}\right)-\text{median}\left(I_{ambient}\right) \end{array}$$ (1)

If the subtracted colour did not represent a real colour within the colourspace to which it was being transformed for analysis, no data was recorded for that particular colourspace.

**Sclera white balancing** The sclera has high specular reflectivity due to the overlying film of lacrimation, and is therefore likely to contain regions of specular reflection which accurately reflect the chromaticity of the illuminant. In order to determine whether this can be used to adjust an image to account for varying ambient lighting, Von Kries white balancing was used as per Eq 2 for each image channel within each region of interest (*I*).

$$\begin{array}{c} I_{whitebalanced}=\text{median}\left(I_{ambient}\right)÷\text{median}\left(I_{ambient,whiteref}\right) \end{array}$$ (2)

where *I*_*ambient*,*whiteref*_ was chosen in four different manners:

1. the 5% of pixels within the sclera with the lowest redness metric,
2. the 15% of pixels within the sclera with the lowest redness metric,
3. the 35% of pixels within the sclera with the lowest redness metric,
4. The 5% of pixels closest to D65 white within the sclera.

For images that did not contain the sclera, *I*_*ambient*,*whiteref*_ was taken from the sclera in a separate image, because the illuminant chromaticity was unlikely to greatly change between the two images.

#### Feature extraction (redness measures)

After the image was adjusted for ambient lighting, potential colour measures were calculated for each region of interest. Consequently, from the preprocessed image, five different classes of features were calculated (erythema index, *r* chromaticity, −*g* chromaticity, a* chromaticity, b* chromaticity), and for each feature, and each region of interest, the median, and median of the top 5% of the feature were taken.

**Feature 1: erythema index**—the erythema index, as per [45], was calculated for the region of interest. It is given by *EI* = *log*(*R*) − *log*(*G*) and is expected to be greater for regions of interest with a higher concentration of haemoglobin, which reflects more red light and absorbs more green light than the surrounding tissue.

Features 2 and 3: a* b* chromaticities—using the D65 whitepoint, the CIE 1976 L*a*b* chromaticity for the region of interest was calculated using the MATLAB conversion function rgb2lab. In this colour-opponent colourspace, (a* = 0, b* = 0) represents a neutral grey. An increase in redness is reflected by an increase in a* whereas green is represented by a decrease in a*. The other colour axis, blue-yellow, is represented by −b* to + b*. In this case, both the a* and b* chromaticity specifiers were used as potential features with which to predict haemoglobin concentration. Due to the fact that a* in particular is strongly influenced by the ratio of red to green, it may therefore be a useful predictor in the same way as erythema index or *r* chromaticity.

**Features 4 and 5: *r*, *g* chromaticities**—the *r* and *g* chromaticities were calculated as *r* = *R*/(*R* + *G* + *B*) and *g* = −*G*/(*R* + *G* + *B*) respectively.

#### Regression and classification

Each feature was correlated against blood haemoglobin concentration using least-squares linear regression. For each region, the best measure was selected by first selecting all measures for which the correlation was statistically significant when compared to a static model (F-test, at *α* = 0.1, followed by Bonferroni correction for multiple statistical tests), and then choosing the remaining measure with the highest *R* value. Multilinear regression was carried out by testing all permutations of pipelines which had a correlation with significance under *α* = 0.01. Each permutation contained one pipeline from each region being incorporated. The optimum combination of features (by correlation coefficient *R*) was selected.

Classification was carried out using a naïve Bayes classifier. The feature input to the classifier was chosen by testing all permutations of pipelines which had a correlation with significance under *α* = 0.01. Each permutation contained one pipeline from each region being incorporated, and the optimum was selected by classification accuracy. For evaluating the results of the selected techniques for classification, leave-one-out validation was used to estimate performance on unseen data; for each patient, a classifier was trained using all patients except the target patient. The trained classifier was then used to predict the unseen target patient, and their classification recorded. The optimum classification parameters were selected using Youden’s J index.

## Results

**Fig 2:**
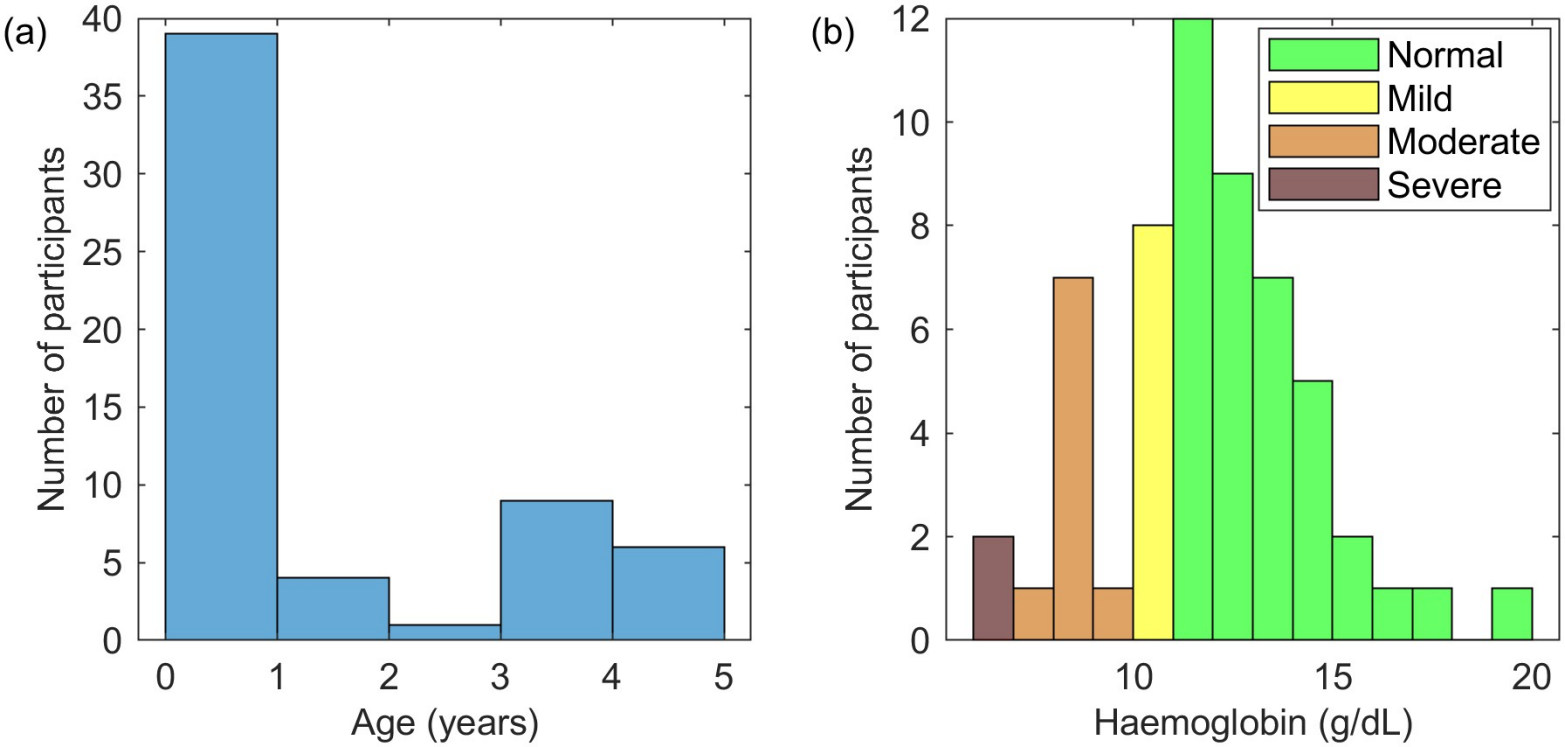
Demographic features of the dataset. (a) shows the age of the participants, at the time the images were taken; (b) shows the measured blood haemoglobin concentration for the participants, colour coded according to the WHO anaemia diagnostic categories, where normal had blood haemoglobin concentration ≥ 11.0 g/dL, mild anaemia was ≥ 10.0 and < 11.0 g/dL, moderate anaemia was ≥ 7.0 and < 10.0 g/dL, and severe anaemia was < 7.0 g/dL.

Fig 2 shows the distribution of the collected data, prior to any exclusions based on image quality. The mean age of participants was approximately 1.25 years (standard deviation 1.65 years, median 0.25 years). The mean concentration of haemoglobin measured in the blood was 11.7 g/dL (standard deviation 2.48 g/dL, median 11.7 g/dL). At a 5% significance level, the measured blood haemoglobin concentrations were approximately normally distributed (Kolmogorov-Smirnov test, *p* = 0.304, *KS* = 0.121).

### Performance of individual ROIs

**Fig 3:**
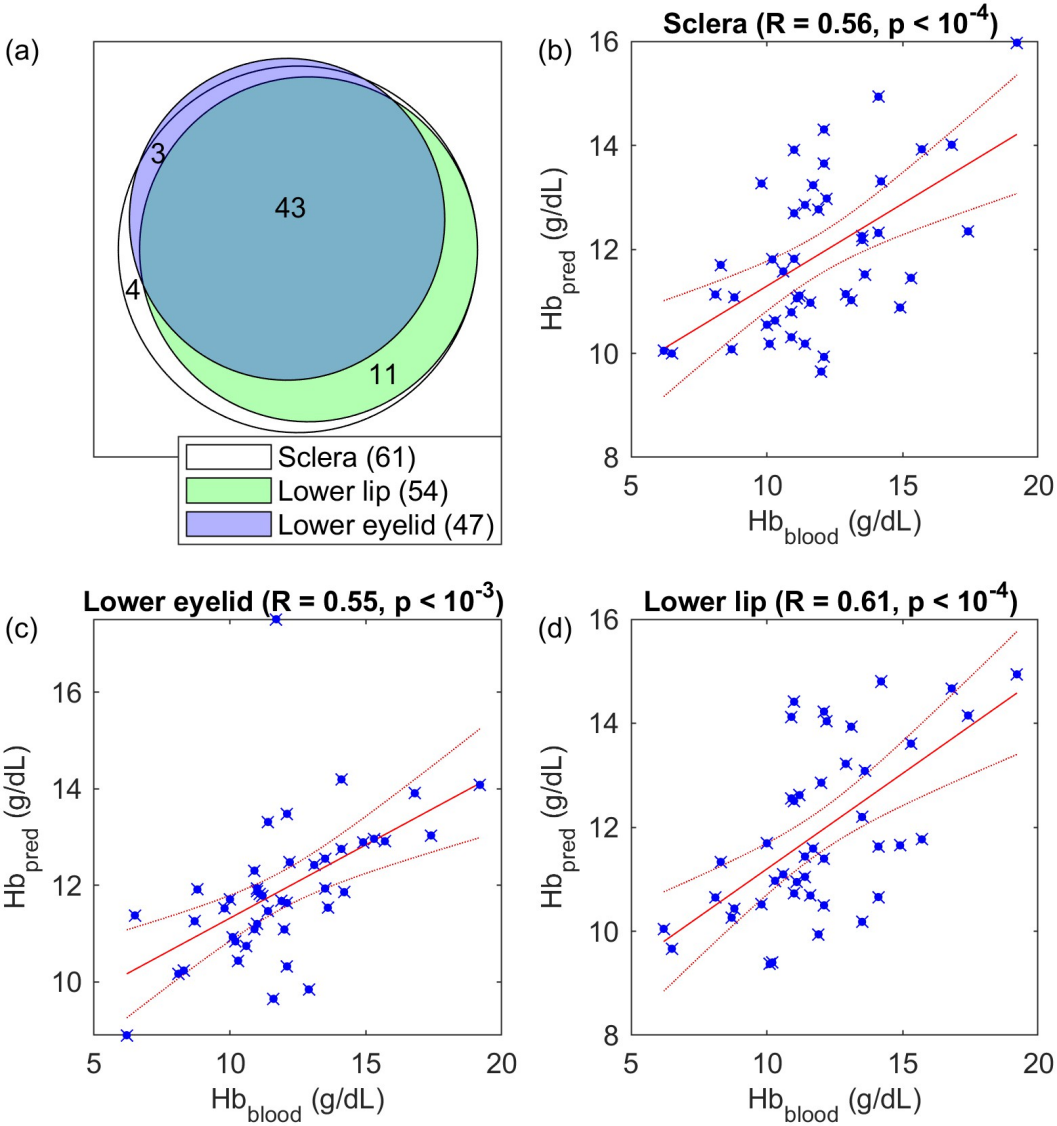
Correlation of extracted region-of-interest chromaticity statistics with measured blood haemoglobin concentration, for the 43 patients with good-quality images for all regions. Red dotted lines show 95% confidence intervals of the line of best fit. (a) number of patients with images for each region of interest; (b) the sclera alone; (c) the lower eyelid conjunctiva alone; (d) the lower lip alone.

**Fig 4:**
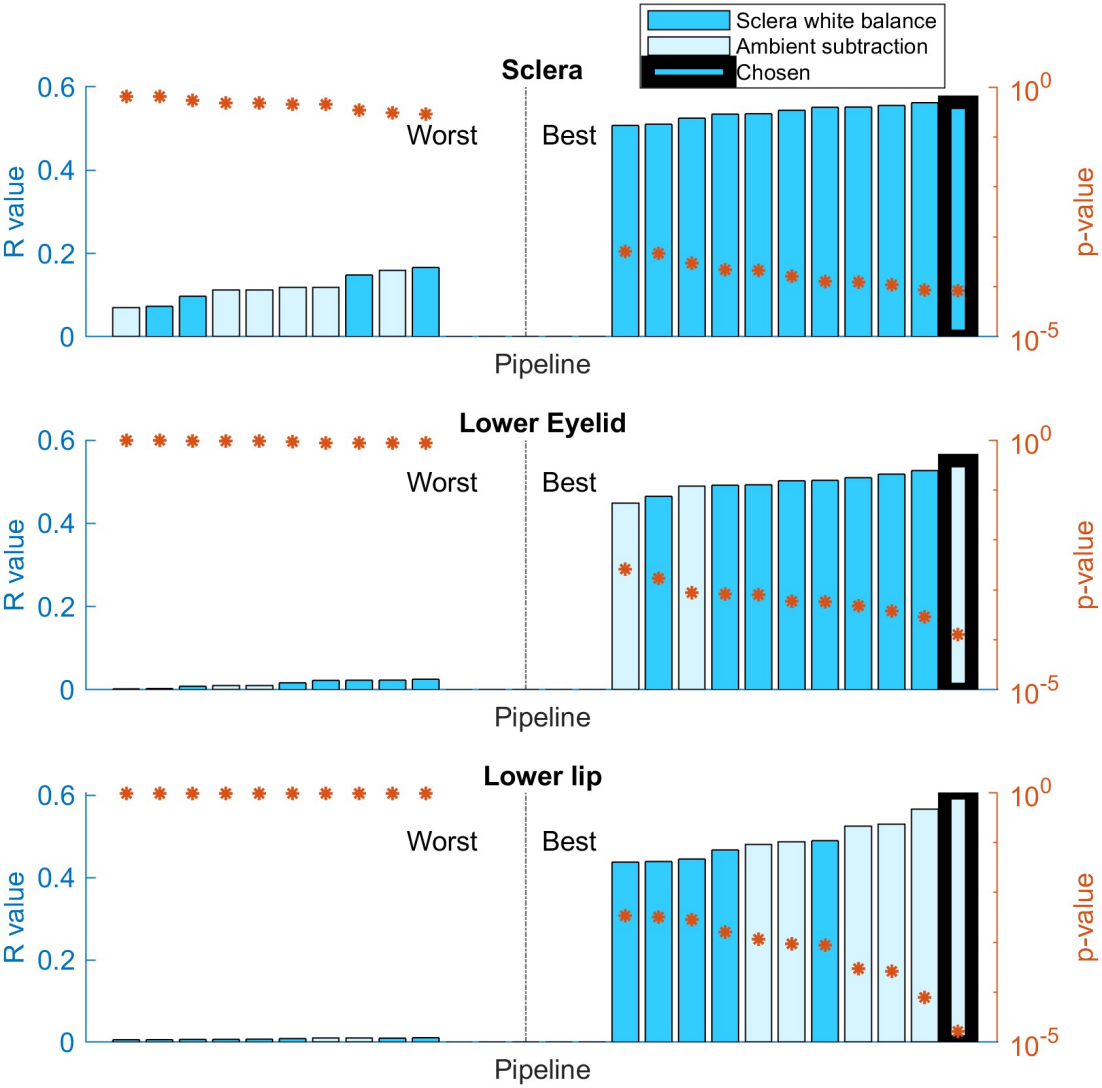
The ten best and worst tested analysis pipelines for each region of interest. Each integer on the x-axis represents a different pipeline. The bar charts show the correlation coefficient (*R*) for the model, the scatter chart shows the p-value for the model. Bars in light blue used ambient subtraction to account for ambient lighting, whereas bars in dark blue used sclera white balancing. Each panel shows a different region of interest: (a) sclera; (b) lower eyelid conjunctiva; (c) lower lip. The optimal pipeline for each region of interest is highlighted with a thicker black bar.

For 19 patients, images of some regions of interest were not collected or not of sufficient quality (e.g. excessively blurry). Panel (a) of Fig 3 shows this in more detail. For the patient images who remained, the mean subtracted signal-to-noise ratio (SSNR, as per [42]) was 5.70. After low quality images were excluded, the SSNR differed significantly between the different regions of interest (one-way ANOVA, *F* = 6.70, *df* = 2, *p* = 1.72 × 10^−3^). Results for the best and worst pipelines tested in each region of interest were investigated, as shown in Fig 4. Three-way ANOVA showed that the subtraction method (*F* = 0.275, *df* = 1, *p* = 0.601) did not significantly affect the correlation of the pipelines, whereas the choice of redness measure (*F* = 14.5, *df* = 4, *p* = 1.04 × 10^−10^) and the region of interest (*F* = 34.6, *df* = 2, *p* = 4.64 × 10^−14^) had a significant effect on the correlation coefficient of the model. On average, the best redness measure was *g* chromaticity, and the best region of interest was the sclera.

Out of the 270 predictors which were investigated, 15 had statistically significant correlations against measured blood haemoglobin concentration (*α* < 0.1, after Bonferroni correction *α* < 3.70 × 10^−4^). Using only the patients for whom images from all regions were present, the optimum predictor of blood haemoglobin concentration for each region of interest was selected and the best regression models for each region plotted as per Fig 3. The results are summarized in Table 2.

**Table 2:** Summary of preliminary results.

Region	Best Preprocessing	Best Redness Metric	R	*p*
Sclera	White balancing (pixels near D65, no flash)	Top 5% by *r*-chromaticity	0.563	8.43 × 10^−5^
Lower eyelid	Ambient subtraction with reddest 5%	Top 5% by *r* chromaticity	0.551	1.28 × 10^−4^
Lower lip	Ambient subtraction with reddest 5%	Top 5% by erythema index	0.606	1.63 × 10^−5^

Best preprocessing technique and redness metric (“feature”) for each region of interest. The correlation coefficient and shown is for linear regression against blood haemoglobin concentration as measured using the HemoCue device.

### Multi-ROI model

**Fig 5:**
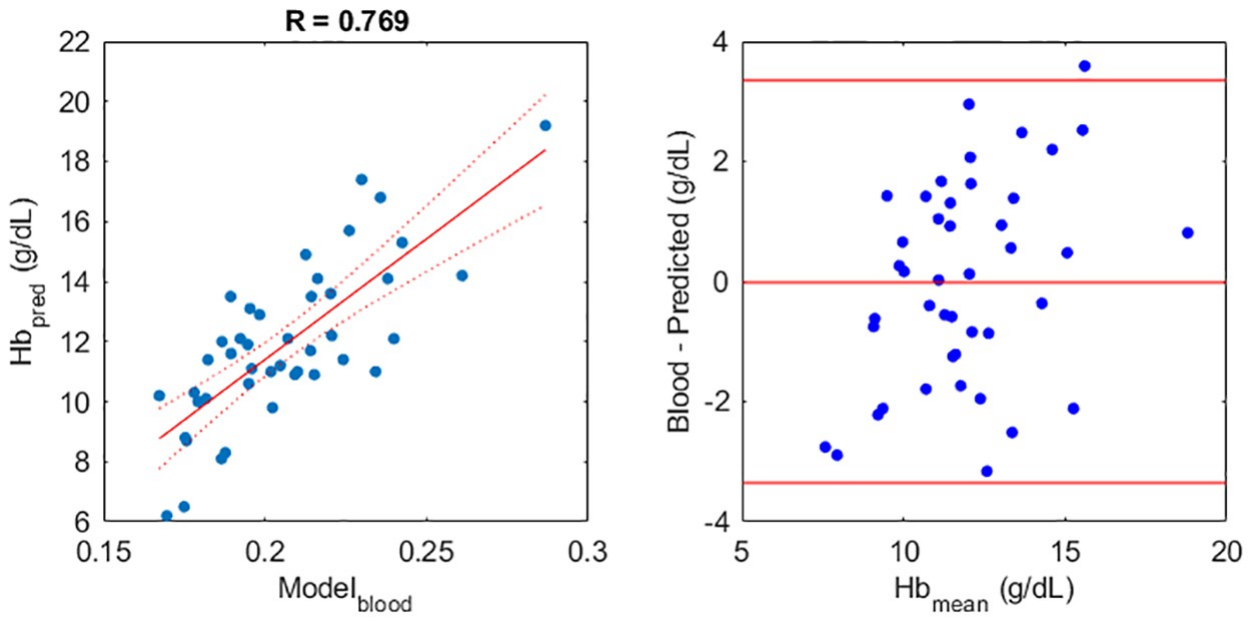
Performance of a multivariate linear model, using the best predictors from the sclera, lower lip, and lower eyelid conjunctiva together; (a) correlation between the predicted haemoglobin concentration, and the measured blood haemoglobin concentration, with 95% confidence intervals for the line shown in dotted red; (b) Bland-Altman analysis of the prediction accuracy. The horizontal lines are the 95% limits of agreement (+/- 3.36 g/dL), and the mean difference (∼0 g/dL).

The best combination of pipelines with one feature from each region of interest was automatically selected. This analysis used the same feature as Table 2 for the sclera. The feature for the lip was now the median of the top 5% of *r* chromaticities, with ambient subtraction based on the top 5% of pixels by redness. The feature from the eyelid was now the median of the top 5% of *g* chromaticities after correction using white balancing of the flash image, with the whitepoint chromaticity taken from the 15% of pixels on the sclera with the lowest redness. The results are shown in Fig 5. The 95% limits of agreement were ±3.36 g/dL.

### Anaemia screening model

The results of naïve Bayes classifiers to classify for anaemia are shown in Table 3. All regions of interest alone had a better accuracy than chance (67.4%) when using leave-one-out cross validation. The mean accuracy of methods using two regions of interest (80.7%) was greater than the mean accuracy when using a single region of interest (70.5%). This accuracy was further increased to 90.7% when all three regions of interest were used together. The three region model had a sensitivity of 92.9% (95% CI from 66.1% to 99.8%). The specificity of the model using the sclera, lower eyelid, and lower lip together was 89.7% (95% CI from 72.7% to 97.8%).

**Table 3:** Summary of preliminary results.

	Random	Sclera	Eyelid	Lip	Sclera + Eyelid	Sclera + Lip	Eyelid + Lip	Sclera + Lip + Eyelid
True Positive	1	6	6	7	9	10	12	13
False Positive	1	4	6	5	7	2	5	3
False Negative	13	8	8	7	5	4	2	1
True Negative	28	25	23	24	22	27	24	26
Sensitivity	7.14%	42.9%	42.9%	50.0%	64.3%	71.4%	85.7%	92.9%
Specificity	96.6%	86.2%	79.3%	82.8%	75.9%	93.1%	82.8%	89.7%
Accuracy	67.4%	72.1%	67.4%	72.1%	72.1%	86.1%	83.7%	90.7%

Performance of naïve Bayes classifiers using different regions of interest to screen for anaemia (blood haemoglobin concentration < 11.0 g/dL). Random prediction was estimated using a Monte-Carlo technique. It is the average of 5000 naïve Bayes classifiers using a single randomly generated input feature each time. 67.4% of the patients in the dataset did not have anaemia. The 67.4% accuracy at random chance represents what would be expected on average if 67.4% of patients were predicted as non-anaemic.

**Fig 6:**
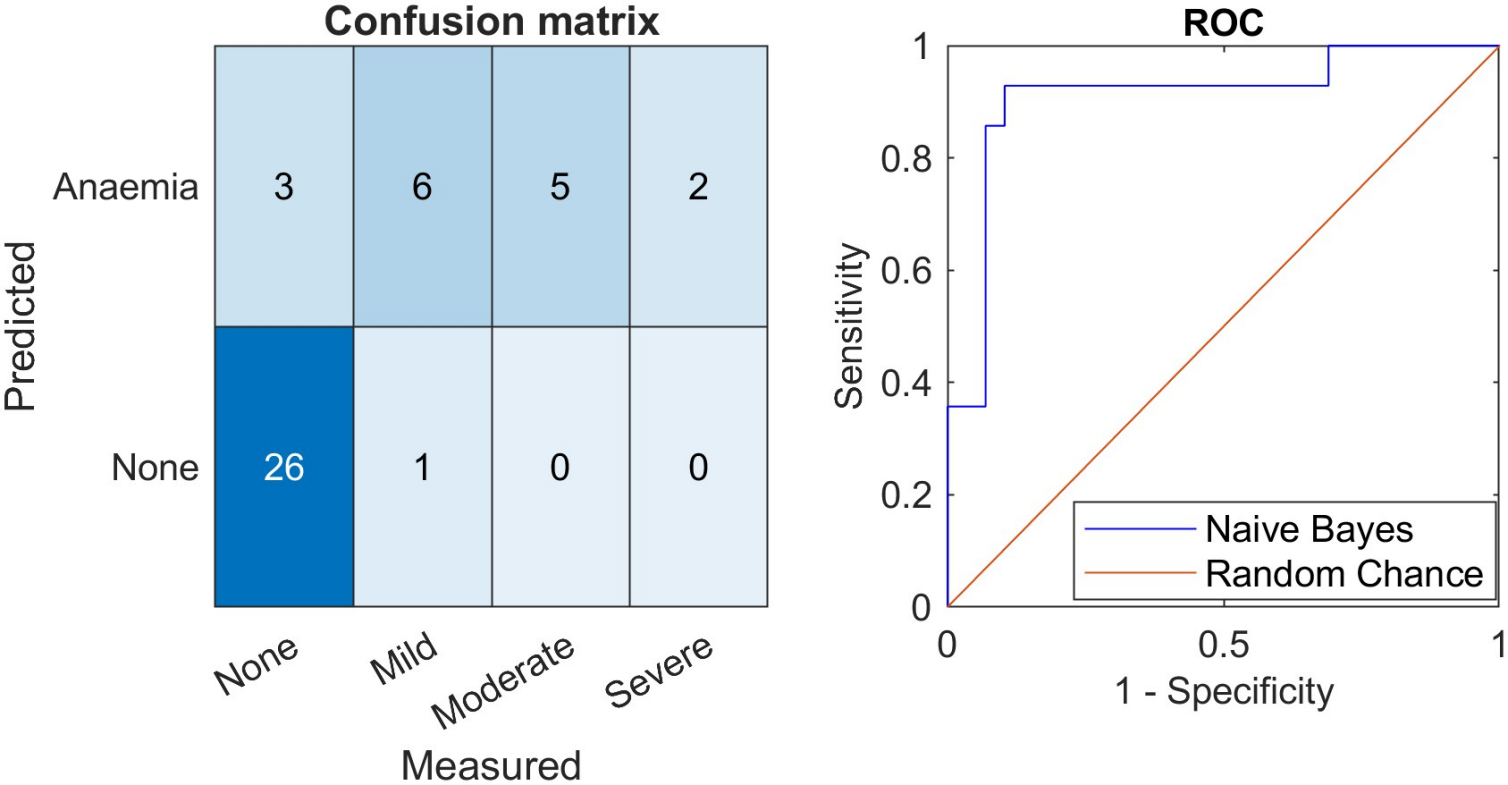
Performance of a naïve Bayes classifier using three regions of interest to screen for anaemia. **Left**: confusion matrix, the performance of the differing models at varying measured blood haemoglobin concentrations. **Right**: ROC curves for identifying participants with haemoglobin concentration <11.0 g/dL, with chance level indicated by the red line, and the present classifier indicated by the blue line. The area under the curve was 0.909.

The optimum model for the three regions of interest used whitebalancing on the no-flash image for the sclera, and ambient subtraction (using points close to D65 white) for the eyelid and lip. For all regions of interest, the median of the top 5% of the redness metric was taken. For the sclera, the redness metric was CIE 1976 b* chromaticity, for the eyelid it was erythema index, and for the lip it was *r* chromaticity. Fig 6 shows a detailed breakdown of performance during cross validation for the model using the sclera, lower lip, and lower eyelid together. Both severely anaemic patients were correctly classified.

## Discussion

This approach has two key stages: (1) the processing and extraction of a feature from each region of interest, and (2) the conversion of these features into a predictor of anaemia. The performance of the present technique demonstrates a reasonable sensitivity and specificity, especially when three regions of interest are used together. As a screening tool, the sensitivity of 92.9% compares well to the sensitivity of 85.0% reported for the HemoCue [47]—but the HemoCue which was used as a gold standard reference in this study is already likely to perform worse than traditional screening methods [48]—and consequently it is, at present, unlikely to be a tool which replaces established clinical practice. However, the sensitivity could allow pre-screening of large amounts of the population at low cost. Such individuals could then be further investigated. The early evidence suggests this screening approach is likely to be relatively low-risk because for the severely anaemic patients, the sensitivity was 100%, only decreasing for less anaemic patients, meaning that very ill individuals are less likely to be missed. Previous work has indicated that similar techniques tend to overestimate low haemoglobin concentrations [26, 39], but if this performance is maintained in a larger sample size, this could be a clinically useful tool. Before this could happen, several limitations must be overcome. This section discuses several conclusions from the results in this paper, and details the major limitations.

### Choosing an analysis pipeline

Two different areas of the analysis pipeline were investigated in this research. The first was the adjustment for ambient lighting, and the second was the extraction of a suitable redness measure. In adjusting for ambient lighting, this study notes that using ambient subtraction led to several additional exclusions due to low subtracted signal-to-noise ratios, which, despite being high on average, occasionally fell below 3.4, the threshold suggested for reduced accuracy [42]. This was partially due to the decision to add a diffuser in front of the smartphone flash, to reduce the brightness and specular reflection from the flash and therefore avoid photosensitivity or discomfort in the children. Overall, sclera white balancing tended to feature as the best pipeline for the sclera, whereas ambient subtraction was more frequently featured in the best predictor from the conjunctiva and the lower lip. It is possible that variations in lighting chromaticity over the scene (e.g. the impact of reflections from the photographer and surroundings) meant that sclera white balancing was only accurate for white balancing of the sclera itself, and not for areas further away from it.

For all three regions of interest, the median of the top 5% of pixels (by redness metric) proved to be the best predictor (although the exact redness metric that was chosen varied between regions). In conjunction with examination of the selected regions, this indicates that the algorithm was effectively performing crude vessel filtering by limiting analysis to the reddest areas. It had originally been hypothesized that this would only be required for the sclera and potentially lower eyelid, because the lower lip, with significant arterial blood flow causing pronounced vermilion [49], may not benefit from filtering due to the density of the blood vessels. Indeed, previous work which measured lip erythema index as a proxy for tissue oxygenation used the erythema index of the entire lip, without filtering to vessels [50]. Similarly [31], used images of the fingertip, illuminated by a bright phone torch; in their study, the scattering and diffusion provided by the deep fingertip meant that the majority of the image was affected by the chromaticity of the blood, and no vessel filtering was required to obtain their results. However, in this analysis case, choosing the 5% of most red pixels was consistently optimal. This may be due to the fact that, as well as filtering for vessels, choosing the top 5% of most red pixels also excludes other confounding areas (such as ulcerations, which may appear less red).

The main novelty of this work was the use of a multi-region model which incorporated of the lower lip as a region of interest. Future work may improve upon this by investigating the effect of taking multiple captures of the same region of interest, in order to reduce “noise” from the environment or phone camera sensor.

### Barriers to translation and adoption

The present method provides a proof of concept for binary screening for the presence or absence of anaemia, which was carried out using a single smartphone. However, it is clinically desirable to know not just whether a patient has, but also how severe that anaemia is. The present dataset did not contain large numbers of severely anaemic patients (just 2 patients). This meant that predicting the severity of anaemia was not feasible. Future work could collect data from more severely anaemic patients. Such a study might be improved by the use of automated techniques for segmentation, in order to reduce interobserver variation between segmentations. These variations in segmentation boundaries due to varying observers would be a potential source of error in the real world, as they may lead to varying amounts of surrounding tissue being included, which would affect colorimetric accuracy. Automated segmentation using a U-Net like architecture has been shown to perform well for the sclera [51] and may therefore be one method through which interobserver variability can be reduced.

Analytical barriers are not the only barriers to adoption of this technique. Even with predictive performance which is clinically useful and validated more widely, there are requirements for appropriate safeguards around the presentation of results. This would include the communication of uncertainty—for which several techniques have been suggested in clinical machine learning [52]. This work is made challenging by the fact that unsophisticated communication of uncertainty (e.g. as simple numerical ranges) can negatively affect understanding and perceived credibility of data [53]. This is compounded by the fact that—even though device independent calibrations are shown to work well [42]—uncertainty may still vary depending upon the characteristics of the smartphone camera and optics. Considering these wider human-computer-interaction factors is key to mitigating the safety challenges of introducing a predictive tool with a significant potential to impact clinical care.

## Conclusion

This paper presents a comparison of different methods for using smartphone imaging for screening for anaemia in a vulnerable and understudied paediatric population. The aim of this paper was to determine which analysis methods would perform best in this population.

We found that the best technique to use for accounting for ambient lighting depended upon which area of the body was being analysed—white balancing using a reference point on the sclera worked well when analysing the sclera, but ambient subtraction performed better elsewhere. The best redness measure with which to predict blood haemoglobin concentration was either the erythema index, CIE 1976 b* chromaticity, or the *r* chromaticity, depending on the region of interest. In all investigated regions of interest—where blood vessels are relatively sparse and there is no transillumination—carrying out pseudo-“vessel filtering” (not using the entire region of interest) by taking the reddest 5% of pixels within the region helped to improve the results over taking the median of all the pixels within the region of interest. This indicates that some, but not all, parameters for colorimetric analysis can be shared between different regions of interest.

Using combinations of different regions of interest together improved the predictive ability. When the lower eyelid, the lower lip, and the sclera are used together, screening performance reaches a level which may be useful for real-world community screening, especially in asymptomatic groups. Using these three regions together means that two image pairs must be captured, but delivers a substantial increase in sensitivity.

The optimal algorithm was able to obtain a sensitivity of 92.9% and a specificity of 89.7% when screening for anaemia as defined by the World Health Organization guidelines. This adds to the body of evidence suggesting smartphones may be useful for anaemia screening. However, barriers around inclusion of different populations and presentation of results must be overcome before such techniques can be translated into medical devices.

## Supporting information

S1 Checklist Inclusivity in global research checklist. (PDF)

S1 Data Anonymized image dataset. Colorimetric features extracted from the participant images. Each row represents a participant, and columns represent a feature. The features are described in the labels in the first row of the document.
(XLSX)

S2 Data Participant metadata. The measured blood haemoglobin concentration and additional information about the participants, collected in order to investigate potential confounding factors or associations.
(XLSX)
